# HeadUp: A Low-Cost Solution for Tracking Head Movement of Children with Cerebral Palsy Using IMU

**DOI:** 10.3390/s21238148

**Published:** 2021-12-06

**Authors:** Sana Sabah Al-azzawi, Siavash Khaksar, Emad Khdhair Hadi, Himanshu Agrawal, Iain Murray

**Affiliations:** 1 SRT Department, EISLAB, Luleå University of Technology, 97187 Luleå, Sweden; 2 College of Engineering, University of Information Technology and Communications, Baghdad 10013, Iraq; 3 School of Electrical Engineering, Computing and Mathematical Sciences, Curtin University, Bentley, WA 6102, Australia; siavash.khaksar@curtin.edu.au (S.K.); himanshu.himanshu@curtin.edu.au (H.A.); i.murray@curtin.edu.au (I.M.); 4 Rehabilitation Medical Center and Joint Diseases, Baghdad 10001, Iraq; mrc_baghdad@yahoo.com

**Keywords:** head control, cerebral palsy, rehabilitation, inertial measurement unit, head movement measurement, health, disability, sensor fusion algorithm

## Abstract

Cerebral palsy (CP) is a common reason for human motor ability limitations caused before birth, through infancy or early childhood. Poor head control is one of the most important problems in children with level IV CP and level V CP, which can affect many aspects of children’s lives. The current visual assessment method for measuring head control ability and cervical range of motion (CROM) lacks accuracy and reliability. In this paper, a HeadUp system that is based on a low-cost, 9-axis, inertial measurement unit (IMU) is proposed to capture and evaluate the head control ability for children with CP. The proposed system wirelessly measures CROM in frontal, sagittal, and transverse planes during ordinary life activities. The system is designed to provide real-time, bidirectional communication with an Euler-based, sensor fusion algorithm (SFA) to estimate the head orientation and its control ability tracking. The experimental results for the proposed SFA show high accuracy in noise reduction with faster system response. The system is clinically tested on five typically developing children and five children with CP (age range: 2–5 years). The proposed HeadUp system can be implemented as a head control trainer in an entertaining way to motivate the child with CP to keep their head up.

## 1. Introduction

Cerebral palsy (CP) is a group of disorders in the developmental milestone, including posture and motor function, that become evident through infancy or early childhood  [1,2]. The CP prevalence ranges from 1.5 to more than 4 per 1000 live births. These statistics are expected to be much higher in developing countries due to low standards of medical care [3].

**Figure 1:**
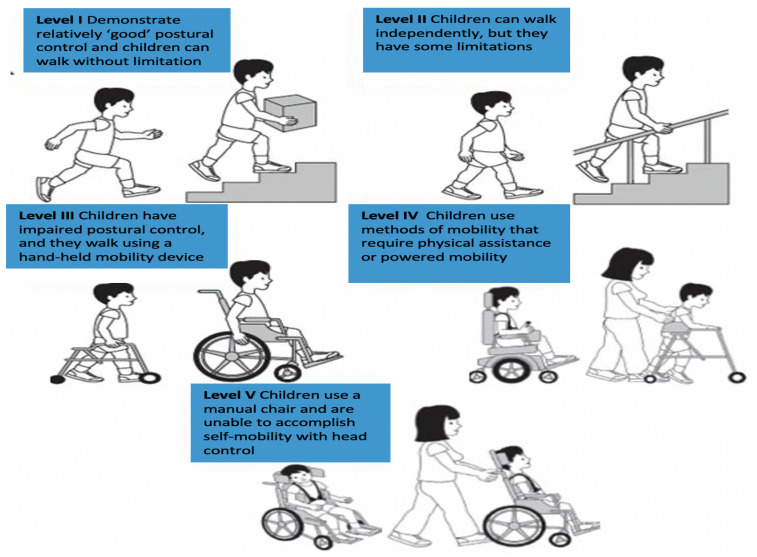
Cerebral palsy levels.

CP is a non-progressive brain disorder, and most children with CP experience spasticity, motor disorders, and a lack of selective motor control [4]. Although CP is permanent, its outcomes can be minimized [5]. These motor difficulties differ from one CP child to another CP child based on the severity level. The Gross Motor Function Classification System (GMFCS)[6] classifies children with CP into five levels, as shown in Figure 1.

Children with CP levels IV-V suffer from one of the essential cerebral palsy problems: poor head control [7].

Head control refers to the ability to control the head upright above the shoulder with respect to gravity while sitting, standing, or walking with a rotation ability in the desired direction. Head stability gives a stable reference to vertical posture. Additionally, poor head control can affect many aspects of children’s lives, such as eating, self-care, self-entertainment, vocational sitting [8,9], and self-esteem [10].

Multiple reasons make the assessment of head motion and head control ability (HCA) a vital study area in the rehabilitation and bioengineering speciality. The most significant reason is helping clinicians evaluate, diagnose, and provide optimal care and treatment for children with CP [11,12]. Moreover, difficulties encountered while preserving head control can be one of the first indications that a child has a development problem.

However, the current diagnosis for poor head control depends on visual inspection [13], which relies on the clinician’s experience and the time he has spent with a patient. Sometimes, clinicians and physiotherapists cannot spend an adequate amount of time assessing HCA. Although parents spend much time with their children, they still cannot provide continuous monitoring for head movement. There is also the problem that people living in remote areas do not have access to medical experts.

Additionally, different studies reported poor accuracy and reliability of visual assessment for joint movement. These studies stated the need for more accurate and reliable tools combined with a visual evaluation to improve examination quality [14,15]. As a result, there is a need for a precise system that can capture head movement and discover the inability to fully control the vertical orientation of the head. There is also a need to provide the best physiotherapy program, to avoid the implications of inaccurate diagnoses and to fully utilize this program.

Inertial measurement unit (IMU) sensors can capture an object’s motion without the need for external reference, cameras, emitters or environmental lighting [16]. The IMU consists of a 3-axis accelerometer, 3-axis gyroscope, and 3-axis magnetometer. There are many clinical applications for IMUs in human movement monitoring [17,18,19,20,21,22].

This paper presents a novel, IMU-based system for HCA tracking that targets children with CP aged from 2 to 5 years old. HeadUp is a head-mounted device that is designed to be small (3×3×3×6.5) cm, that is easy to wear, that is lightweight (<50 g), and that consumes low power. Additionally, the device’s raw data are available and could be accessed easily for future analysis to identify the required physiotherapy program. Moreover, the proposed device is designed to be inexpensive in contrast to other existing systems and specifically for head movement monitoring. The most important challenge is extracting medical information from the raw system’s readings to assess the HCA of the child and this study will address this challenge in Section 6.

The main contribution of this paper is the use of a custom built and low-cost sensor for capturing head movement of children with CP in the age range of 2 to 5 years. Most of the research in the literature target older children (5–12 years) with either higher CP levels such as level I or II, where the child already has better motor ability and reasonably good head control [23,24,25]. The paper shows the application of a low-cost engineering solution to capture clinically viable data which allows clinical professionals and physiotherapists to get a better understanding of the active range of head movement for children with CP. Anecdotal feedback from the physiotherapists has been positive since there are very limited methods for capturing the active range of movement, especially for the age range of 2 to 5 years.

The remainder of this study has been constructed as follows: related work is presented in Section 2. System design is described in Section 3. This section includes the HeadUp device design and the filter algorithm to filter the measured data’s noise. System implementation is explained in Section 4. Section 5 presents the results and Section 6 discusses the results. Section 7 concludes the paper and presents future work.

## 2. Related Work

Head movement or cervical movement is the ability to smoothly and accurately move the head to a given pattern. There are several possible methods to measure head movement. Head motion measurement systems or cervical range of motion (CROM) have been gradually adopted in the medical profession for many purposes. Different methods have been employed to measure head motion [26].

An ancient study was conducted in 1962 [27], in which the author reviewed the various methods for joint motion measurement in general and head motion measurement in particular, such as protractors with arms, optical goniometers, and pendulum goniometers. These measurements made it difficult to accurately determine head motion. Many recent studies have discussed more accurate and trustworthy methods for head movement measurement [28,29,30,31,32].

One of the best and most accurate methods is an optical motion capture system [32] that uses cameras and optoelectronic markers to track head movement. Although this method is convenient, such systems are easily affected by occlusion and lighting effects, which tend to reduce the system efficiency. Additionally, such systems are costly and complex to implement in laboratories [33]. Another issue is the difficulty of a testing environment for small children.

Different systems have been proposed to overcome these limitations. IMUs showed excellent implementation in the field of three-dimensional motion analysis [17,34,35,36]. In [36], Rudigkeit et al. compared different commercially available IMU-based systems under various conditions to control an object by head movement. The authors reviewed the control model presented in the literature and highlighted the advantages and disadvantages of each method. All previously mentioned methods can be an effective way to measure the head control ability HCA of children with cerebral palsy.

Many researchers have investigated some of these methods for training children with CP to achieve better head control [11,37,38], especially between 1970 and 1990 [7,39,40,41,42]. Harris et al. [7] developed an electronic device that can be placed in an oversized helmet to monitor head movement while converting this movement to electrical signals, providing auditory guidance to achieve an upright position. The authors tested the effectiveness of the head control device (HCD) on nine children with CP, with ages ranging from 7 to 18; all of these children improved their head stability after using the device from approximately a few seconds to more than 5 min in duration of holding a fixed posture. Harris et al. did not provide any statistical details about the enhancement after treatments, and the degree of improvement for each patient is unknown.

Subsequently, mercury tilt switches were employed to detect the head deviation from the specified angle range [39,40,41]. In [39], a mercury tilt switch was installed on an earphone to turn a transistor radio ON and OFF to reinforce head posture for two children with CP whose ages ranged from 9 and 17 years old. Radio music was activated when the child’s head was in the upright position. The authors discovered that music can promote head control for children with CP. A wearable head position trainer (HPT) was designed in [40] and tested on 12 CP children aged 3–10 years. HPT provided auditory feedback and number count of head position deviations beyond a given angle for each child. A large number of researchers have applied the HPT in their studies [41,42,43].

Although these studies stated an improvement in HCA, many drawbacks and limitations in their devices are noted [44]. Some of these disadvantages are described as follows:

- These devices were heavy (220 g without the helmet), and some of the children experienced difficulty raising their head while wearing the apparatus compared with those without wearing the device.
- Cables must connect these devices to a control unit, increasing the size of the overall system.
- These devices did not provide any information about head movement in 3 dimensions.
- The presence of a physiotherapist is mandatory during the experiments.
- Difficulty in reliable device positioning occurred because of the working principle of mercury tilt switching.
- A deviation angle threshold for each child had to be established and applied every time the device was used.

Unfortunately, there are few recent studies concerning head movement and head control ability tracking for children with CP. In 2018, head motion was measured for children with CP using a video-based approach. The authors placed markers on the ear and temporal fossa, using cameras to record head movement. This approach has a beneficial effect on HCA assessment [11]. The same approach was employed [38] to estimate the head orientation. Even though this approach solves the previously mentioned limitations and difficulties, it is costly and difficult to set by students.

The head motion tracking system presented in this paper is inexpensive, easy to wear, and designed specifically for head motion monitoring of CP to provide valuable information for therapists about HCA.

## 3. Methodology

### 3.1. HeadUp System Design

**Figure 2:**
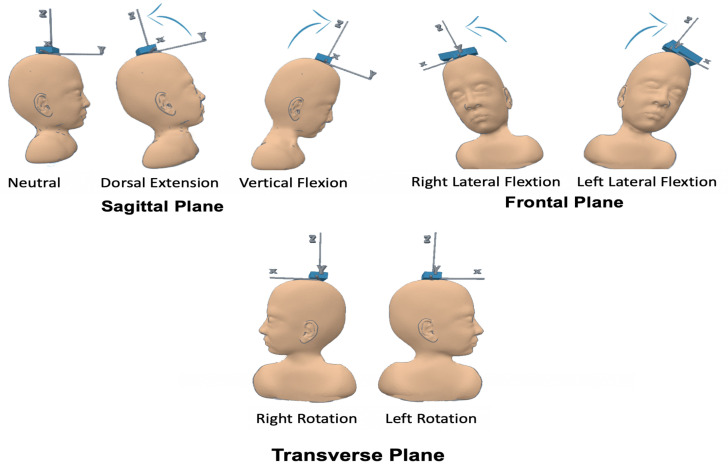
Head motion terms.

The proposed HeadUp system was designed and tested as a medical HCA evaluation system. The HeadUp device is constructed to be convenient for patients who wear it and to be informative for physicians who use it to monitor the parameters of the patient’s head movements. As shown in Figure 2, head motion can be described in the following terms [45]:

- Flexion (F)
- Extension (E)
- Right Lateral Flexion (RLF)
- Left Lateral Flexion (LLF)
- Right Rotation (RR)
- Left Rotation (LR)

**Figure 3:**
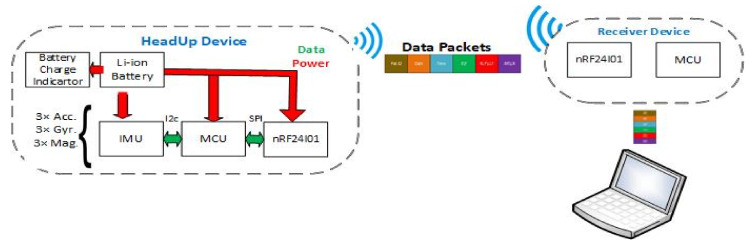
HeadUp system architecture.

These head motion parameters are essential for evaluating the HCA of patients with CP. As illustrated in Figure 3, the HeadUp system includes the HeadUp device and the receiver device.

The HeadUp device comprises a microcontroller (Arduino Pro Mini 3.3 V), a 9-axis IMU sensor (MPU9255), a charge indicator unit, and transceivers (nRF24L01). The MPU9255 has a 3-axis accelerometer (Acc), 3-axis gyroscope (Gyro), and 3-axis magnetometer (Mag). The receiver device includes another microcontroller and nRF24l01 transceiver to receive the data. Both the HeadUp device and receiver device were powered by a rechargeable 1000 mAh Livion 3.7 battery. The total cost of the HeadUp system (HeadUp and receiver dongle) was approximately 18$.

**Figure 4:**
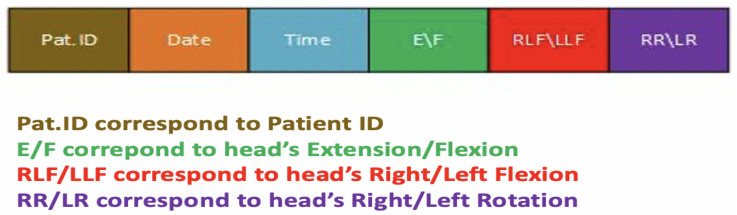
HeadUp data packet.

**Figure 5:**
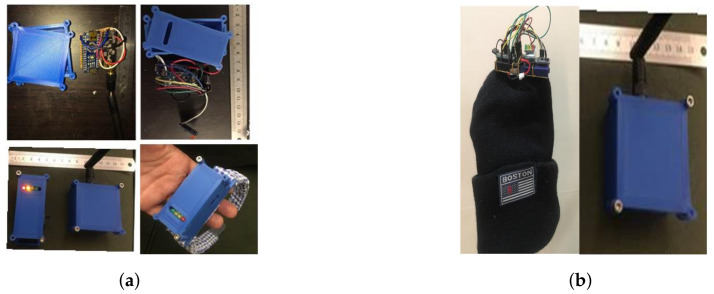
HeadUp system (transmitter and receiver). (**a**) HeadUp for moderate CP. (**b**) HeadUp for severe CP.

The microcontroller, which is the main component for the HeadUp device, collects data by an inter-integrated circuit protocol ($I^{2}C$) bus, calculates the head movement parameters, encapsulates the data into packets with patient Id, dates, and sends the packets to the receiver device by radio-frequency. The data shown in Figure 4 will be collected by the receiver device and sent to a laptop through the UART protocol. The data are then saved in a (CSV) file for further analysis. The HeadUp system (transmitter and receiver devices are shown in Figure 5.

### 3.2. Data Acquisition and Sensor Fusion Algorithm

#### 3.2.1. Data Acquisition

First, the raw data were obtained while the HeadUp device were stable; these raw data could not be applied without calibration. All three sensors (Acc+Gyro+Mag) need to be calibrated, and two values must be measured for each sensor (bias and scale). Bias is the difference between the raw value and the zeroes, while the scale value represents how much larger the range of sensor data is than the actual value of the physical movement. Without calibration, the measurements lack accuracy. Therefore, calibration is mandatory before the device is utilized.

The IMU sensor was sampled at a 100 Hz frequency and 16-bit resolution. The desired measurement from the HeadUp device is HCA, which can be evaluated by CROM angle measurement (θ,ϕ,ψ). These measurements were obtained by using the raw data from the IMU sensor.

The Acc measures the acceleration based on Newton’s second law and the associated force. The head movement angles (extension/flexion (θ) and right/left lateral flexion (ϕ)) can be measured from only the calibrated Acc measurements using the following equations:

(1) $$\theta_{a}=\tan^{-1}\frac{a_{x}}{{\left(a_{x}\right)}^{2}+{\left(a_{z}\right)}^{2}}\times \frac{180}{\pi }$$

(2) $$\phi_{a}=\tan^{-1}\frac{a_{y}}{{\left(a_{y}\right)}^{2}+{\left(a_{z}\right)}^{2}}\times \frac{180}{\pi }$$

where $\theta_{a}$ is the estimated E/F angle and $\phi_{a}$ is the estimated RLF/LLF angle using only the Acc readings; $a_{x},a_{y},a_{z}$ are the acceleration along *x*, *y*, and *z* axis.

**Figure 6:**
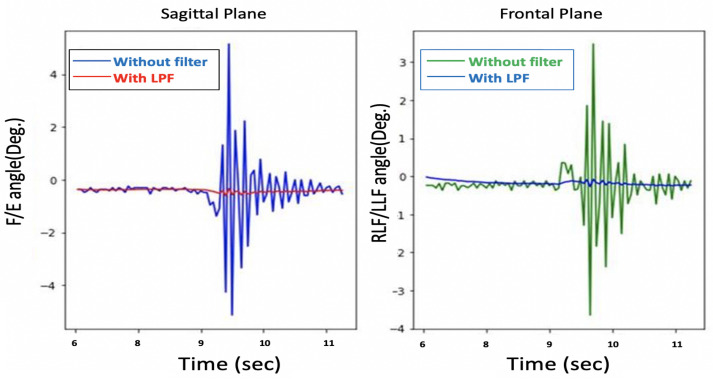
Before and after low pass filter with only Acc.

The problem with these head movement angles, which are measured only from the Acc, is that they are susceptible to vibration and suffer from noise, as shown in Figure 6. A Butterworth low pass filter (LPF) was selected to filter the noise. A Fourier transform was used to calculate the cut-off frequency for the LPF.

**Figure 7:**
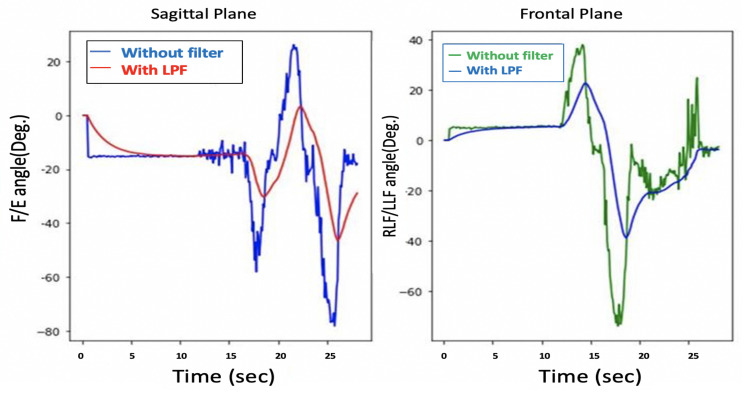
θ and ϕ before and after LPF with only Acc.

**Figure 8:**
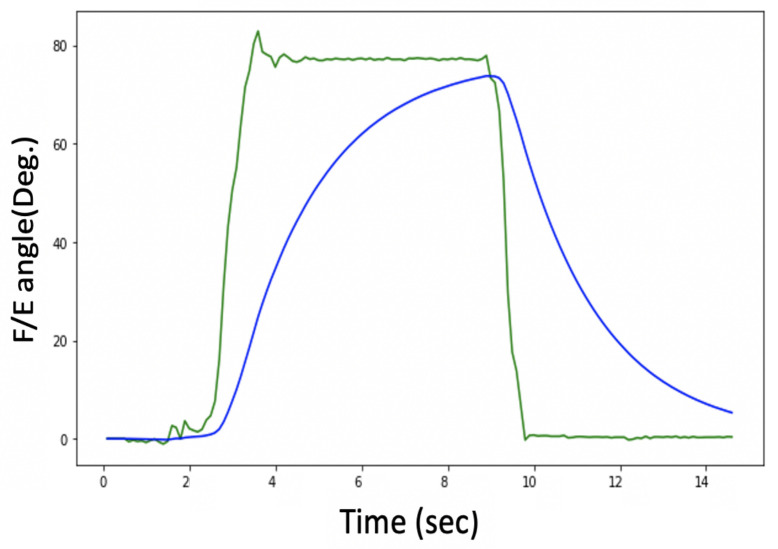
Delay problem with Acc. readings.

Although the filtered θ and ϕ were noise-free, as shown in Figure 7, the system response is sluggish, as depicted in Figure 8.

Figure 8 shows an estimation of the F/E angles, and the lag in the measurement can be clearly seen, so there is a trade-off between getting a noise-free signal and the system’s response time.

**Figure 9:**
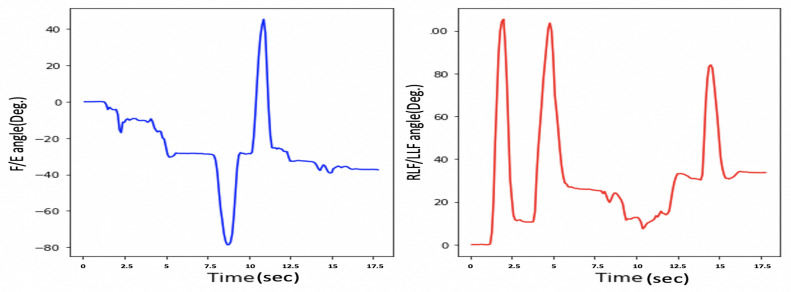
Drift problem in only gyroscope.

Another option for measuring head movement is to use a Gyro. The Gyro measures the object’s angular velocity and can also indirectly measure head movement θ and ϕ by integrating angular velocity over time using the following equation:

(3) $$\theta_{g}\left(t+\Delta \right)=\theta_{g}+\omega_{y}\Delta t$$

(4) $$\phi_{g}\left(t+\Delta \right)=\phi_{g}+\omega_{x}\Delta t$$

where $\theta_{g}\left(t+\Delta \right)$ and $\theta_{g}$ are the new and previous estimated E/F angles using only the Gyro readings. $\phi_{g}\left(t+\Delta \right)$ and $\phi_{g}$ are the new and previous estimated RLF/LLF angles using only the Gyro readings; $w_{x},w_{y},w_{z}$ which are the rotational velocities around *x*, *y*, and *z* axis. The issue with the Gyro readings is that the $\theta_{g}$ and $\phi_{g}$ drift over time, as illustrated in Figure 9. This figure reveals that the head flexion drifts approximately 30${}^{{}^{\circ}}$ after 5 s. Both noise and drift contribute to unacceptable results.

We can conclude that neither the Acc nor the Gyro provide accurate head movement measurements. Therefore, a sensor fusion algorithm (SFA) was implemented to overcome each sensor drawback and to obtain a noise-free and fast system response.

#### 3.2.2. Sensor Fusion Algorithm

As mentioned in Section 3.2.1, the readings from the Acc are not accurate for high-frequency situations such as fast movement in short time intervals; that is why Gyro readings are used for these instances. To obtain accurate head movement measurements, an SFA was implemented to overcome the drawbacks of using each sensor alone and to get reliable readings and improve overall accuracy. To get faster response time and noise-free signal, a complementary filter was used by combining the desired low-frequency characteristic of the Acc and the desired high-frequency characteristic of the Gyro, as shown in Equations (5) and (6).

(5) $$\theta \left(t+\Delta \right)=\left(1-\alpha \right)\left[\theta \left(t\right)+\omega_{y}\Delta t\right]+\alpha \theta_{a}$$

(6) $$\phi \left(t+\Delta \right)=\left(1-\alpha \right)\left[\phi \left(t\right)+\omega_{x}\Delta t\right]+\alpha \phi_{a}$$

where θ(t+Δ) and θ are the new and previous estimated E/F angles. ϕ(t+Δ) and ϕ are the new and previous estimated RLF/LLF angles. α is a constant (0<α<1). The larger the α the more Gyro is trusted, and as α converging to zero, we base our measurement more and more on the Acc readings. For the HeadUp system we chose α to be =0.95.

Equations (5) and (6) put a high-pass filter on the Gyro measurements and a low pass filter on the Acc measurements, and then these signals are combined depending on the constant α to form the final angle estimation.

The complementary filter comprises of a high pass filter and a low pass filter and it may be applicable when the data received from the Acc and Gyro has errors at some period of time. On the one hand, small external forces can create errors in the measurements of the Acc, which means they are not reliable in the short term. On the other hand, the Gyro usually provides accurate data in a short time frame before the drift error can cause problems. To solve these issues, a low-pass filter can be utilized for the Acc, while a high-pass filter can be applied to the Gyro data [46]. As both low- and high-pass filters are included in the complementary filter, high-frequency components and low-frequency components can be handled. The fused data from the sensors will contain much less error compared to individual sensor readings.

Another filtering method that tends to be more complex is called Kalman filtering. The  filter uses a recursive algorithm that can estimate unknown data using historical measurements [46]. There are several stages in the Kalman filter method. First, an initial value will be acquired from the system alongside a prediction algorithm used to correlate a prediction error [47]. Once a new measurement is available, the filter uses it to update the prediction value and the prediction error. As in this project we were aiming to implement algorithms on an embedded Arduino microcontroller with limited computational power, we opted for a method with less computational cost [48], that is complementary filtering.

**Figure 10:**
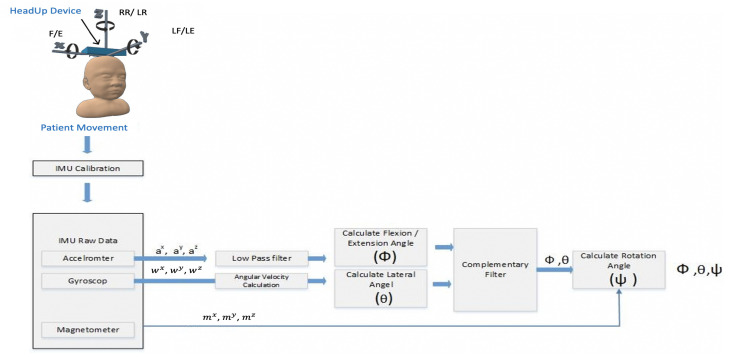
HeadUp SFA and filtering procedure.

The Acc and Gyro can only measure the head F/E and head RLF/LLF. A magnetometer is needed to measure the head’s right/left rotation; ψ as illustrated in Equation (2). The applied SFA is presented in Figure 10; the steps are shown in Algorithm 1.

(7) $$\psi =\tan^{-1}\frac{m_{y}\cos\phi -m_{z}\sin\phi }{m_{x}\cos\theta +m_{y}\sin\phi \sin\theta +m_{z}\cos\phi \sin\theta }\times \frac{180}{\pi }$$

where ψ is the estimated RR/LR angles and $m_{x},m_{y},m_{z}$ are the magnetometer raw readings; strength of the earth’s magnetic vector along the sensor’s *x*, *y*, and *z* axis.
**Algorithm 1:** Sensor Fusion Procedure
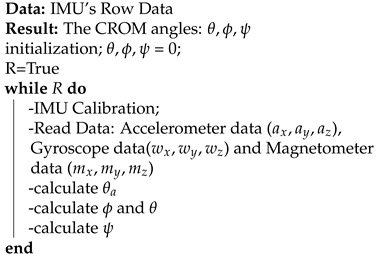


As shown in Figure 10, the SFA has the following steps:

1. Calibration step for all the sensors’ readings (Acc, Gyro, and Mag) to ensure that all the measurements are close to zero when the system is at rest;
2. Butterworth low pass filter for the Acc readings to get rid of high-frequency additive noise;
3. To get faster response time and noise-free measurements, a complementary filter was used;
4. Finally, the magnetometer was used along with Acc and gyro to acquire the head rotation.

**Figure 11:**
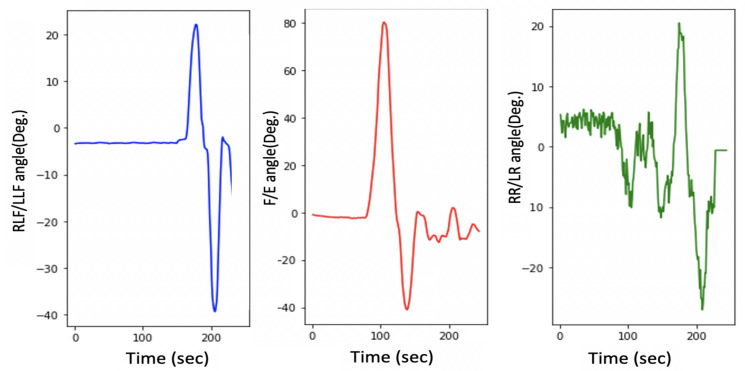
Head movement with a 9-axis IMU for typically developing child.

The measurements from the HeadUp device are presented in Figure 11.

This figure shows the fast response time and the clean measurement with no cumulative drift or noise, demonstrating the proposed SFA’s validity.

### 3.3. System Validation

**Figure 12:**
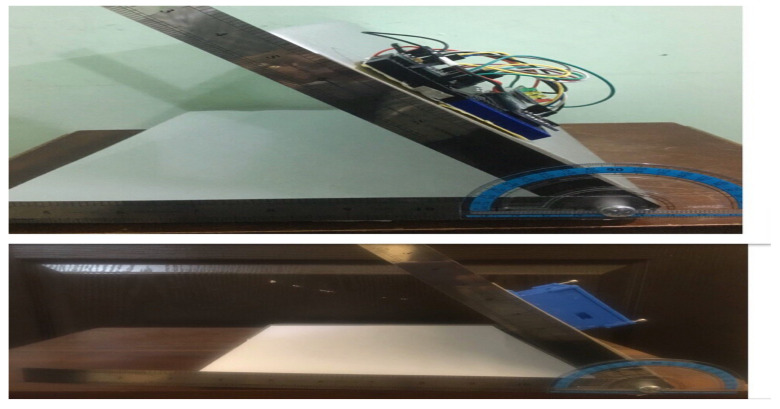
HeadUp device validation using protractor with arm.

**Figure 13:**
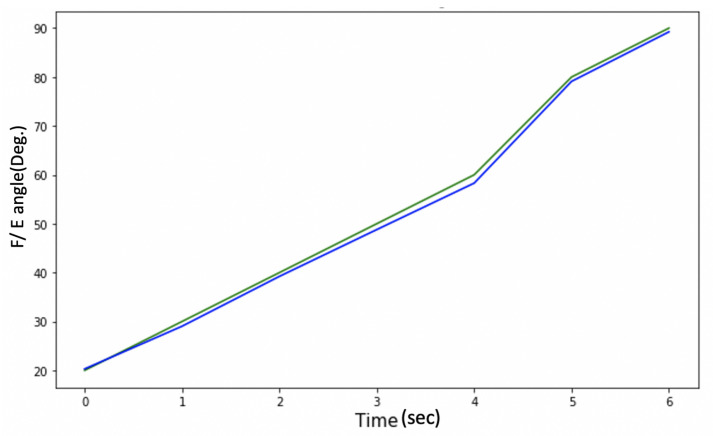
Protractor with arm vs. HeadUp.

The accuracy of the HeadUp device was tested by comparing the flexion angle measurement with another CROM tool in both Sagittal and frontal planes. A protractor with an arm was employed as the validation device, which was composed of two arms with a protractor in between the arms, and could be utilized to give the CROM [49]. This comparison was performed by placing both devices on a flat surface with an adjustable angle to measure the decline of the surface, as presented in Figure 12. The surface was moved at four angles (30${}^{{}^{\circ}}$, 45${}^{{}^{\circ}}$, 80${}^{{}^{\circ}}$, and 90${}^{{}^{\circ}}$). To ensure reading validity, a linear analysis was performed for the CROM measurements recorded by the protractor with the arm and HeadUp device (Figure 13), and the mean differences in the measurement from both devices were calculated. Note that the proposed SFA for the HeadUp device will filter all the noise and fix the drift problem if there is any as explained in Section 3.2. The validation results are discussed in Section 5.

## 4. System Implementation

### 4.1. Subjects’ Selection

Ten male and female children have participated in this research (five children with various CP degrees (male: 4; female: 1; age: 2–5)); This age range was selected because it is a significant period to train the HCA (early age) for future improvement, and the definitive diagnosis of CP is difficult before two years of age [50]. All the selected subjects with CP suffered from poor HCA (level IV-V), responded to simple tasks, and understood straightforward instructions. Five typically developing children were also selected for this research with the same age range (2–5 y/o) [5].

Table 1 shows the characteristics of the participants with CP; the clinicians usually asked for these data during the assessment.For the typically developed children, all of them were healthy.

### 4.2. Measurements

As mentioned in Section 3.1, the HeadUp device includes an IMU sensor consisting of a 3-axis gyroscope, 3-axis accelerometer, and 3-axis magnetometer As previously mentioned, the main aim of this study is the use of custom build sensors to help capture active range of head movement for children with CP (level V and IV) and help control their movements. In such CP levels, the child cannot walk or stand or sit by him/herself, the child can only sit with support. This means the lower part of the body will be stationary and will not affect head movement, as a result only one IMU has been used to capture the head movement. Additionally, different studies showed that one IMU can capture the full head orientation [24,51,52,53].

**Figure 14:**
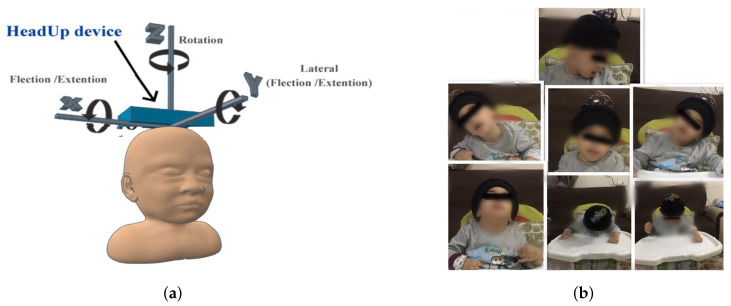
System implementation. (**a**) HeadUp placement. (**b**) Child with CP wearing HeadUp device.

A HeadUp device was mounted on the subject’s head using a head cap for severe children with CP, while it was located on a hair hoop for children with mild CP. Figure 14 shows that HeadUp was mounted on the child’s head, allowing the child to move his/her head in different directions while seated. HeadUp was used to gather the acceleration, magnetic field, and angular velocity of the head. The data gathered from HeadUp were applied to evaluate the HCA based on the proposed method.

Each child wore the HeadUp device while sitting with lumbar support and was instructed to perform the following movement (refer to Figure 2) [24,54,55]

- Case 1: sit still without any movement (Natural).
- Case 2: dorsal extension and ventral flexion.
- Case 3: right lateral flexion and left lateral flexion.
- Case 4: right rotation and left rotation.

These movements are recorded by the receiver device, which receives the data packets (Figure 4), and displays the results on the host computer and saves the data as a CSV file. The HeadUp system measured head angular movement (sagittal plane, frontal plane) and rotational movement (transverse plane) at 100 Hz.

## 5. Result

**Figure 15:**
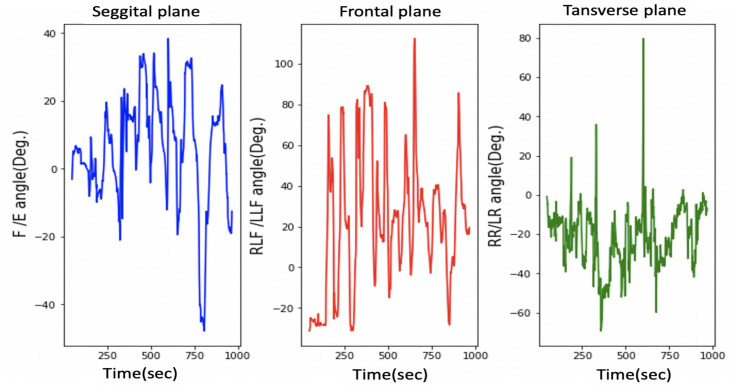
Head movement in 3 dimensions for a child with CP.

As mentioned in Section 1, HCA affects many aspects of a child’s life. Head stability gives a stable reference to vertical posture, which is very important to achieve the vertical posture. Figure 15 illustrates the HCA in the sagittal plane, frontal plane, and transverse plane during the sitting trial for subject 1 (child with CP). Figure 11 shows the same head movement for a typically developing child of the same age and no reported CROM problem.

Figure 11 and Figure 15 show the same sequence head movements; the subjects were asked to do F/E, LLF/RLF, and RR/LR head movement. Figure 11 shows that head movements for a typically developed child. That figure clearly indicates the child could perform these moves, and he/she has a good head control ability and no CROM problem. While Figure 15 indicates that the child with cerebral palsy had trouble controlling their head movement and that figure contains more random movement than Figure 15.

**Figure 16:**
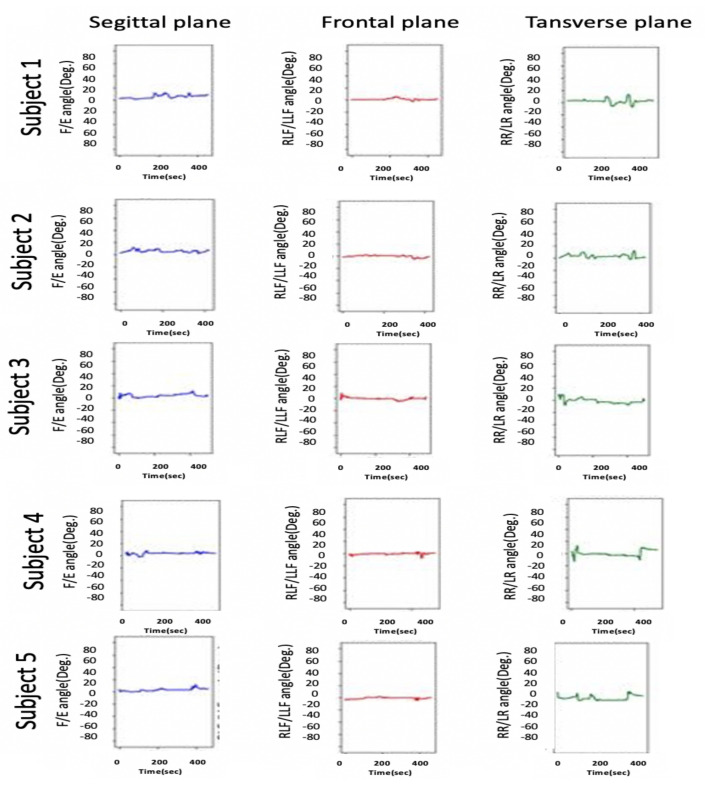
Head movement for typically developing children.

**Figure 17:**
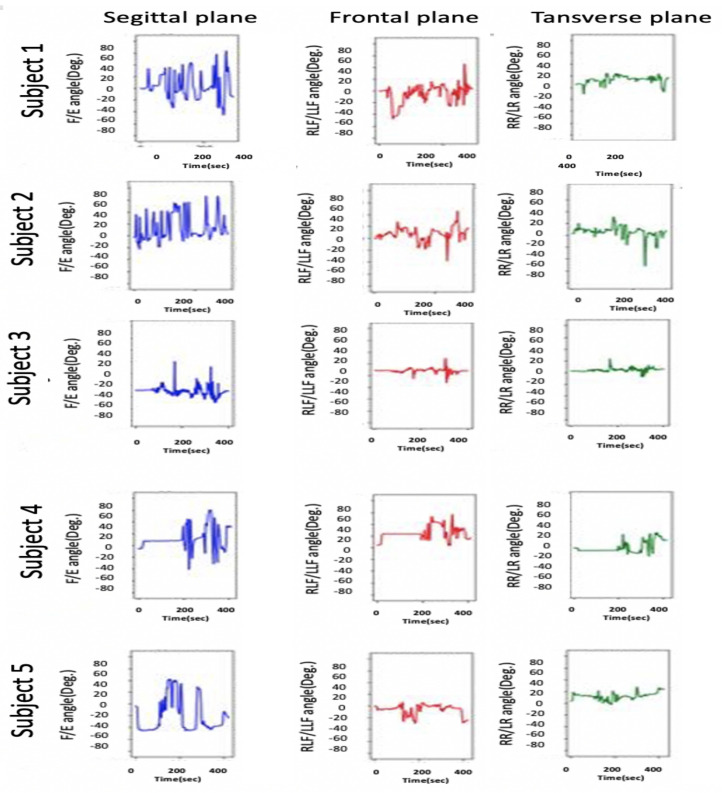
Head movement for children with CP.

The research experiments start with case 1, where all participants were asked to sit without any head movement. Figure 16 and Figure 17 shows the results for case 1. The experimental results for each participant show considerable differences among the head movement signals captured from typically developing participants and those captured from children with CP.

As illustrated in Figure 17 in the sagittal plane for children with CP, continuous variation in the F/E angle is missing for normal children, whereas normal head control in the sagittal plan can be observed in Figure 16. Additionally, this difference is noted on the RLF/LLF in the frontal plane in both figures. Figure 17 indicates the poor HCA for children with CP.

Moreover, Figure 17 shows if the child’s head and trunk rotated or collapsed consistently to one side. This information helps the physiotherapist perform physical therapy for each child with CP.

Note that the head control ability for subject 1 was tested in different ways, the results of which are depicted in Figure 15 and Figure 17. Figure 15 depicts the case where the instructions were to perform a different head movement, and that figure shows the child’s poor ability to control his head. Then Figure 17 shows the same subject trying to sit still without any head movement. The subject was able to control his head rotation in the Transverse plane, but in the Sagittal plane and Frontal plane, it is hard for him to hold his head.

The second part of the study experiments consisted of cases 2, 3, and 4, where all participants were asked to perform simple head movements (F, E, LLF, RLF, RR, and LR). The results for this part are shown in Table 2.

As mentioned in Section 3.3, the device accuracy was tested against another CROM tool; protractor with arm. The mean square error was calculated to see how close the HeadUp’s measurements were to the ground truth. The reported mean square error was less than 2${}^{{}^{\circ}}$, which demonstrates the validity of the HeadUp device. Although the test was done for only 6 s, device validity was tested for a longer time during research experiments. Figure 11 and Figure 15, Figure 16 and Figure 17 show the fast response time and the clean readings with no accumulative drift or noise, demonstrating the proposed SFA’s validity.

## 6. Discussion

This study showed an investigation in applying an IMU-based system to capture active head movement and measure head control ability for young children with severe CP; level IV or V. It was found that the HeadUp system demonstrated the capability to record the head movement in E/F, LLF/RLF, and RR/LR while sitting.

Moreover, HeadUp device allows physicians to capture children’s active head movement while they are engaged in various activities—whether in the health center or throughout their regular lives in their home. The HeadUp system could aid in improving diagnostic and in the suggestions for treatments.

The HeadUp device was mounted on the subject’s head while sitting with lumbar support, as illustrated in Figure 14, and all the participants were instructed to perform different head movements.

Figure 16 and Figure 17 show the HCA without any instructed activity. In normal cases, the head movement angles should be around zero, and this can be clearly seen in Figure 16.

Figure 16 shows that the HeadUp devise is able to estimate the head movements for typically developing children in all directions. The difference in the sagittal plane in Figure 16 and Figure 17 indicates the number of head drops for children with CP. This shows the lack of head control ability by children with CP which has been captured by the sensor. Figure 17 shows the HCA for children with CP. Different medical information can be extracted from that figure:

1. All participants with CP have a poor head control ability.
2. The head of subject 4 collapsed consistently to the right side, while the head of subject 5 collapsed consistently to the left side, which mean the muscles in these sides are weak(this can be seen from RLF/LLF angles).
3. Subject 3 has better HCA than other participants with CP.

After the first stage, different instructions were given to the participants to evaluate the ability of HeadUp system to measure the range of motion (ROM), as shown in Table 2. There were several noticeable differences between the ROM of typically developing children and children with CP. For example, the average ROM in the transverse plane (LR and RR) for a child with CP was 63${}^{{}^{\circ}}$ compared with an average ROM of 79${}^{{}^{\circ}}$ for typically developing children. This means that the head rotation ability for children with CP is lower than Typically developed children.

Head motion in the frontal plane for normal children had a larger mean ROM (35${}^{{}^{\circ}}$ RLF and 35${}^{{}^{\circ}}$ LLF) for the five healthy participants compared with a mean of 32${}^{{}^{\circ}}$ RLF and 33${}^{{}^{\circ}}$ LLF) for the children with CP. The mean CROM in the sagittal plane (Flexion) was 60${}^{{}^{\circ}}$ for normal children, while it was 55${}^{{}^{\circ}}$ for children with CP. All these medical information can be valuable for improving diagnostic and in the suggestions for treatments and physiothereby program.

## 7. Conclusions

Head movement tracking for children with CP with proper efficiency is a challenge. There is a need for a low-cost, accurate, and easy-to-use device that can evaluate and analyze the HCA to detect poor head control for children with CP. Therefore, this research paper presents in-house head motion monitoring to analyze the HCA and to provide the best physiotherapy program.

On the basis of the acquired results, the proposed HeadUp device showed an average reliability of 2${}^{{}^{\circ}}$ compared with other CROM tools (protractor with arm). The HeadUp-based IMU device captured head movement in three planes (sagittal, frontal, and transverse). An SFA was presented to overcome the high noise in the measurements, the noise was reduced in the filtered readings, and as a result, the accuracy of the measurements was increased, which paved the way to accurately identify the HCA for children with cerebral palsy. IMU utilization resolves the problems faced by current CROM methods, such as accuracy, expense, and size, by designing a portable, small, and low-cost alternative that clinicians and physiotherapists can utilize for monitoring the HCA to identify head movement abnormalities.

The validity and reliability of the HeadUp device against one of the standard head angle measurement tools (protractor with arm) were evaluated in two planes (sagittal and frontal). HeadUp’s evaluation gives therapists important information that helps in identifying the appropriate and effective rehabilitation program. In future work, several possible improvements to the system can be explored further, such as:

- Validates the HeadUp system’s results against a more reliable tool in three planes with both devices on the child’s head.
- Investigates different filtering algorithms, such as the Kalman filter
- Use the HeadUp device in an entertaining way as a HCA trainer and examine its performance to improve the child’s head stability.
- Constructed a standalone HCA diagnosis device with the help of machine learning algorithms to distinguish head movement disorder from the typical head movement patterns pattern.

## Figures and Tables

**Table 1:** Participants’ with CP characteristics.

No.	Name	Age	Sex	CP Level	Test Condition	Notes
1	IM	3.5	M	V	sitting	full term baby, mild squint, Mixed Cp
2	AH	5	M	V	sitting	full term baby, sever squint
3	MA	2	M	IV	sitting	full term baby, No squint
4	FH	5	F	V	sitting	full term baby, mild squint
5	HJ	3.5	M	IV	sitting	neglected baby

**Table 2:** Mean values and standard deviations of CROM for the participants.

Movements	Typically Developing Children	Children with CP
F	60.3${}^{{}^{\circ}}$ ± 13.30${}^{{}^{\circ}}$	55.4${}^{{}^{\circ}}$ ± 10.11${}^{{}^{\circ}}$
E	30.38${}^{{}^{\circ}}$ ± 9.10${}^{{}^{\circ}}$	25.22${}^{{}^{\circ}}$ ± 10.40${}^{{}^{\circ}}$
RLF	35.7${}^{{}^{\circ}}$ ± 6.98${}^{{}^{\circ}}$	32.1${}^{{}^{\circ}}$ ± 7.75${}^{{}^{\circ}}$
LLF	35.30 ± 8.30${}^{{}^{\circ}}$	33.81${}^{{}^{\circ}}$ ± 9.82${}^{{}^{\circ}}$
LR	80.04${}^{{}^{\circ}}$ ± 8.03${}^{{}^{\circ}}$	60.96${}^{{}^{\circ}}$ ± 10.23${}^{{}^{\circ}}$
RR	78.81${}^{{}^{\circ}}$ ± 10.86${}^{{}^{\circ}}$	66.03${}^{{}^{\circ}}$ ± 9.27${}^{{}^{\circ}}$

## Data Availability

The data presented in this study are available on request from the corresponding author. The data are not publicly available due to patient privacy.

## Abbreviations

The following abbreviations are employed in this manuscript:

CP	Cerebral palsy
CROM	cervical range of motion
IMU	inertial measurement unit
SFA	sensor fusion algorithm
GMFCS	Gross Motor Function Classification System
HCD	head control device
HPT	head position trainer
F	Flexion
E	Extension
RLF	Right Lateral Flexion
LLF	Left Lateral Flexion
RR	Right Rotation
LR	Left Rotation
Acc	accelerometer
Gyro	gyroscope
LPF	low pass filter
HCA	head control ability
ROM	range of motion
